# Trends in Guideline-Concordant Care for Inflammatory Breast Cancer

**DOI:** 10.1001/jamanetworkopen.2024.54506

**Published:** 2025-02-26

**Authors:** Audree Tadros, Brian Diskin, Varadan Sevilimedu, Amy Xu, Perri Vingan, Jonas Nelson, Yoshiko Iwai, Monica Morrow, Oluwadamilola M. Fayanju

**Affiliations:** 1 Breast Surgery Service, Department of Surgery, Memorial Sloan Kettering Cancer Center, New York, New York; 2 Now with Saint John’s Cancer Institute, Santa Monica, California; 3 Biostatistics Service, Department of Epidemiology and Biostatistics, Memorial Sloan Kettering Cancer Center, New York, New York; 4 Department of Radiation Oncology, Memorial Sloan Kettering Cancer Center, New York, New York; 5 Plastic and Reconstructive Surgery Service, Department of Surgery, Memorial Sloan Kettering Cancer Center, New York, New York; 6 Department of Surgery, Massachusetts General Hospital, Harvard Medical School, Boston; 7 Division of Breast Surgery, Department of Surgery, Perelman School of Medicine, The University of Pennsylvania, Philadelphia; 8 Rena Rowan Breast Center, Abramson Cancer Center, Penn Medicine, Philadelphia, Pennsylvania; 9 Penn Center for Cancer Care Innovation, Abramson Cancer Center, Philadelphia, Pennsylvania; 10 Leonard Davis Institute of Health Economics, The University of Pennsylvania, Philadelphia

## Abstract

**Importance:**

Inflammatory breast cancer (IBC) is an aggressive variant for which trimodality treatment (ie, neoadjuvant systemic therapy [NST] followed by modified radical mastectomy without immediate reconstruction and postmastectomy radiotherapy [PMRT]) represents guideline-concordant care (GCC) and is associated with improved overall survival (OS). However, it is unclear whether there are disparities in trimodality treatment receipt among patients with IBC and how such disparities might affect OS.

**Objective:**

To assess trends in IBC trimodality treatment receipt in a contemporary cohort.

**Design, Setting, and Participants:**

A retrospective cohort study was conducted using data from the National Cancer Database. Women with nonmetastatic IBC treated from calendar years 2010 to 2018 were included. Data analysis was performed from April 1, 2023, to March 1, 2024.

**Exposures:**

Guideline-concordant care (ie, trimodality treatment administered in the correct sequence with time to NST initiation <60 days post diagnosis).

**Main Outcomes and Measures:**

The main outcomes were associations between patient-, disease-, treatment-, and facility-level factors and receipt of overall and modality-specific GCC and associations between these factors and adjusted OS.

**Results:**

Of 6945 patients identified (median age, 57 [IQR, 47-66] years; 2.4% Asian or Pacific Islander, 7.8% Hispanic, 17.1% non-Hispanic Black, and 71.5% non-Hispanic White), only 1740 (25.1%) received all forms of GCC: 91.3% (n = 5662) received NST initiation less than 60 days post diagnosis, 63.3% (n = 4395) received PMRT, and 51.3% (n = 3564) underwent guideline-concordant surgery (ie, modified radical mastectomy without immediate reconstruction with >6 lymph nodes removed). Receipt of GCC did not differ significantly by race and ethnicity, insurance status, or location. Asian (odds ratio [OR], 0.48; 95% CI, 0.27-0.84), Black (OR, 0.53; 95% CI, 0.41-0.68), and Hispanic (OR, 0.40; 95% CI, 0.29-0.55) patients were less likely to have NST initiation less than 60 days post diagnosis vs White patients (all *P* ≤ .001). Recipients of GCC had improved adjusted OS vs nonrecipients (hazard ratio [HR], 0.75; 95% CI, 0.68-0.84; *P* < .001). Black patients had significantly lower adjusted OS ,compared with White recipients (HR, 1.41; 95% CI, 1.26-1.58; *P* < .001). When GCC was received for triple-negative IBC, there was no racial and ethnic disparity in OS.

**Conclusions and Relevance:**

In this cohort study of women with nonmetastatic IBC, there were no disparities observed in GCC receipt, but only 25.1% of patients with IBC received all forms of GCC for which they were eligible. Among those who received GCC, there was no racial disparity in survival for triple-negative IBC, suggesting opportunities to improve equity through standardization of care.

## Key Points

**Question:**

Does receipt of guideline-concordant care (GCC) for inflammatory breast cancer (IBC) vary over time and across different patient populations?

**Findings:**

In this cohort study of 6945 US women with nonmetastatic IBC, GCC receipt did not by differ significantly by race and ethnicity or insurance status, but only 25% of the patients received all the GCC treatments for which they were eligible. Among GCC recipients, Black patients were 40% more likely to die than White patients, but no racial and ethnic disparity was observed for triple-negative IBC.

**Meaning:**

The findings of this study suggest that receipt of GCC may be especially necessary for racially minoritized patients with IBC.

## Teaser

This cohort study examines factors associated with the receipt of trimodality treatment among women with inflammatory breast carcinoma.

## Introduction

Inflammatory breast carcinoma (IBC) is an aggressive type of breast cancer that accounts for 1% to 5% of cases each year but up to 10% of breast cancer deaths.^1^ Inflammatory breast carcinoma is a clinical diagnosis in which patients develop breast cancer associated with erythema and/or edema of at least one-third of the breast in a short period of time (<6 months).^2^ The diagnosis is often delayed because symptoms can mimic benign conditions, such as infection or inflammation, although guidelines have recently been developed to guide diagnosis.^3^ Timely receipt of treatment is paramount given the rapid onset and progression of this disease.

Over the past 2 decades, overall survival (OS) for patients with nonmetastatic IBC has improved with the use of trimodality therapy^4^ comprising neoadjuvant systemic therapy (NST) followed by modified radical mastectomy without immediate reconstruction and postmastectomy radiotherapy (PMRT).^5,6^ Among patients receiving trimodality treatment, 5-year survival rates have been reported to be 55% and 10-year survival rates have been reported to be 37%.^6^

Black women with IBC have historically had lower survival compared with White women.^7,8,9^ Previous work examining whether variation in treatment might be responsible for this disparity in survival has not yielded consistent evidence of racial treatment disparities. However, most of these studies lack granularity on the quality metrics associated with systemic therapy, surgery, or radiotherapy. Specifically, receipt of trimodality treatment alone does not equate with receipt of guideline-concordant care (GCC). Patients may experience long delays to initiation of systemic therapy or they may receive inappropriate oncologic surgery, as studies have shown increasing rates of immediate (instead of completely delayed) breast reconstruction and sentinel lymph node biopsy (instead of axillary lymph node dissection) among this group of patients, despite a lack of data to support these practices.^10,11,12,13^ Accordingly, we sought to examine longitudinal trends in GCC receipt for patients with IBC, whether GCC receipt varies by race and ethnicity and insurance status, and whether differential GCC receipt may be associated with survival.

## Methods

### Data Source and Study Cohort

The National Cancer Database is a joint effort by the American Cancer Society, the American College of Surgeons, and the Commission on Cancer to analyze and track treatments and outcomes of patients diagnosed with malignant neoplasms and includes more than 70% of the new cancers diagnosed in the US each year. Institutional review board approval for this study was obtained from Memorial Sloan Kettering Cancer Center, and it was deemed exempt due to the use of deidentified data. The study followed the Strengthening the Reporting of Observational Studies in Epidemiology (STROBE) reporting guideline for observational studies.^14^ Data analysis was performed from April 1, 2023, to March 1, 2024.

Women with nonmetastatic IBC (cT4d N0 3M0) treated from calendar year 2010 to 2018 were identified. To capture all patients who did and did not undergo GCC, patients meeting the criteria were included regardless of treatment received. Only patients with early discontinuation of radiotherapy were excluded as this could represent disease progression. Patients were excluded if they had a prior cancer diagnosis.

### Study Variables

The following patient-level data were abstracted: age; race and ethnicity as reported by the National Cancer Database,^15^ which were combined into a single variable; insurance status; and patient location based on the urban and rural continuum. Based on the US Department of Agriculture Economic Research Service 9-level Rural-Urban 2013 continuum code (RUCC), patients residing in RUCC levels 1 to 3 are categorized as living in metropolitan counties, 4 to 7 as urban counties, and 8 to 9 as rural counties.^16^ Tumor characteristics, including clinical and nodal category and stage, treatment facility type, breast and axillary surgery performed, receipt of NST, receipt of PMRT, and pathologic staging after completion of systemic therapy were also abstracted. Guideline-concordant care was defined as trimodality treatment (ie, NST, modified radical mastectomy without reconstruction, and PMRT) administered in the correct sequence. We also examined modality-specific GCC, including time to initiation of NST less than 60 days and guideline-concordant surgery, defined as complete axillary lymph node dissection (>6 lymph nodes removed)^17^ at the time of modified radical mastectomy without reconstruction, and receipt of PMRT in the correct sequence following surgery. Tumor subtype was reported as *ERBB2* (formerly *HER2* or *HER2/neu*) hormone receptor–positive/non-*ERBB2* overexpressing (*ERBB2*-negative), hormone receptor–positive/*ERBB2* overexpressing (*ERBB2*-positive), hormone receptor–negative/*ERBB2*-positive, and triple-negative (hormone-receptor–negative/*ERBB2*-negative).

### Statistical Analysis

Data were compared between patients receiving and not receiving GCC, using χ^2^ tests for categorical variables and *t* tests or nonparametric tests (Wilcoxon rank sum or Kruskal-Wallis test) for continuous variables. Univariate and multivariable regression analyses were performed to examine associations between patient-, disease-, treatment-, and facility-level factors and receipt of both overall and modality-specific GCC.

Overall survival was measured from the time of diagnosis until the time of death or last follow-up. Unadjusted OS was estimated using the Kaplan-Meier method with the log-rank test used to compare groups, and the Cox proportional hazards regression model was performed to estimate the association between patient-, disease-, treatment-, and facility-level factors and adjusted OS. We considered 2-sided values of *P* < .05 to be statistically significant. All statistical analyses were conducted using R, version 4.2 (R Foundation for Statistical Computing).

## Results

### Receipt of GCC 

A total of 6945 women (median age, 57 [IQR, 47-66] years) with nonmetastatic IBC were included. Of the 6726 individuals who had data on race and ethnicity, 522 (7.8%) were Hispanic, 161 (2.4%) were non-Hispanic Asian or Pacific Islander (hereinafter, Asian or Pacific Islander), 1153 (17.1%) were non-Hispanic Black (hereinafter, Black), and 4808 (71.5%) were non-Hispanic White (hereinafter, White) (Table 1). Of the 6945 patients, 3313 were treated between 2010 and 2013 and 3632 were treated between 2014 and 2018. Of the 6836 individuals who had data available on insurance, most patients had private insurance (50.7% [3469]), with 28.7% Medicare (1964), 14.2% Medicaid (974), 5.0% uninsured (339), and 1.3% other government insurance (90). A total of 5589 of 6739 patients (82.9%) resided within a metropolitan geographic location, and most patients were treated at comprehensive community cancer programs (ie, ≥500 newly diagnosed cancer cases accessioned by pathology reports per year; 40.5% [2524 of 6237]) or academic research programs (ie, ≥500 new cancers cases accessioned by pathology reports per year and “participates in postgraduate medical education in at least 4 program areas, including internal medicine and general surgery”^18^; 32.2% [2009 of 6237]). Approximately two-thirds of patients had ductal histologic characteristics: 83.6% (5743 of 6873) were clinically node-positive (cN1-3) at presentation and only 14.4% (999 of 6945) of the patients had a pathologic complete response to NST.

**Table 1.** Patient, Tumor, Treatment, and Facility Characteristics^a^

Characteristic	All patients, No. (column %) (N = 6945)	Patients, No. (row %)	
		Non-GCC (N = 5205)	GCC (N = 1740)
**Patient characteristic**			
Age range, y			
<40	708 (10.2)	535 (75.6)	173 (24.4)
40-49	1390 (20.0)	1048 (75.4)	342 (24.6)
50-59	1998 (28.8)	1444 (72.3)	554 (27.7)
60-69	1595 (23.0)	1145 (71.8)	450 (28.2)
≥70	1254 (18.0)	1033 (82.4)	221 (17.6)
Race and ethnicity			
Asian or Pacific Islander	161 (2.4)	110 (68.3)	51 (31.7)
Black	1153 (17.1)	866 (75.1)	287 (24.9)
Hispanic	522 (7.8)	419 (80.3)	103 (19.7)
White	4808 (71.5)	3579 (74.4)	1229 (25.6)
Other^b^	82 (1.2)	62 (75.6)	20 (24.4)
Unknown	219	169	50
Insurance type			
Medicaid	974 (14.2)	750 (77.0)	224 (23.0)
Medicare	1964 (28.7)	1541 (78.5)	423 (21.5)
Other government	90 (1.3)	68 (75.6)	22 (24.4)
Private/managed care	3469 (50.7)	2493 (71.9)	976 (28.1)
Uninsured	339 (5.0)	267 (78.8)	72 (21.2)
Unknown	109	86	23
Geographic location			
Metropolitan	5589 (82.9)	4234 (75.8)	1355 (24.2)
Rural	148 (2.2)	104 (70.3)	44 (29.7)
Urban	1002 (14.9)	721 (72.0)	281 (28.0)
Unknown	206	146	60
Greater circle distance, miles			
≤10	3102 (50.7)	2375 (76.6)	727 (23.4)
10.1-50.0	2466 (40.3)	1840 (74.6)	626 (25.4)
50.1-100	325 (5.3)	229 (70.5)	96 (29.5)
>100	223 (3.6)	162 (72.6)	61 (27.4)
Unknown	829	599	230
**Tumor characteristic**			
Overall stage			
I	154 (2.6)	111 (72.1)	43 (27.9)
II	1833 (30.7)	1390 (75.8)	443 (24.2)
III	3988 (66.7)	2942 (73.8)	1046 (26.2)
Unknown	970	762	208
Clinical nodal category			
N0	1130 (16.4)	905 (80.1)	225 (19.9)
N1	3649 (53.1)	2681 (73.5)	968 (26.5)
N2	1080 (15.7)	798 (73.9)	282 (26.1)
N3	1014 (14.8)	768 (75.7)	246 (24.3)
Unknown	72	53	19
Pathologic complete response			
Yes	999 (14.4)	728 (72.9)	271 (27.1)
No	5946 (85.6)	4477 (75.3)	1469 (24.7)
Tumor subtype			
Hormone receptor–positive/*ERBB2*-negative	2386 (36.5)	1688 (70.7)	698 (29.3)
Hormone receptor–negative/ *ERBB2*-positive	1189 (18.2)	849 (71.4)	340 (28.6)
Hormone receptor–positive/*ERBB2*-positive	1212 (18.5)	1055 (87.0)	157 (13.0)
Triple negative	1754 (26.8)	1283 (73.1)	471 (26.9)
Unknown	404	330	74
**Treatment characteristics**			
Neoadjuvant systemic therapy given within 60 d of diagnosis			
Yes	5662 (91.3)	3922 (69.3)	1740 (30.7)
No	540 (8.7)	540 (100.0)	0
Unknown	743	743	0
Total mastectomy without immediate reconstruction			
Yes	3564 (51.3)	1824 (51.2)	1740 (48.8)
No	3381 (48.7)	3381 (100.0)	0
Radiotherapy given in proper sequence			
Yes	4395 (63.3)	2655 (60.4)	1740 (39.6)
No	2550 (36.7)	2550 (100.0)	0
**Facility characteristics**			
Academic research program	2009 (32.2)	1502 (74.8)	507 (25.2)
Community cancer program	510 (8.2)	395 (77.5)	115 (22.5)
Comprehensive community cancer program	2524 (40.5)	1896 (75.1)	628 (24.9)
Integrated network cancer program	1194 (19.1)	877 (73.5)	317 (26.5)
Unknown	708	535	173

Abbreviation: GCC, guideline-concordant care.

^a^ Calculation of all percentage values only include patients with nonmissing data.

^b^ Other includes Aleutian or Eskimo, American Indian, Chamorran, Fiji, Guamanian, Hawaiian, Melanesian, Micronesian, New Guinean, Polynesian, Samoan, Tahitian, and Tongan. Race and ethnicity were largely self-reported, but the National Cancer Database contributing sites categorize race per the *Facility Oncology Registry Data Standards *manual, which has specific rules for adjudicating racial assignment including for individuals identifying as biracial or multiracial.^15^

Overall, 1740 patients (25.1%) received GCC and 5205 patients (74.9%) did not receive GCC. Receipt of GCC decreased over time, with 32% of patients receiving GCC in 2010 vs 19% of patients in 2017 (eFigure 1 in Supplement 1). Notably, 45% of patients received GCC between 2010 and 2012 compared with only 22% of patients between 2016 and 2018. A total of 5662 of 6202 patients (91.3%) received NST within 60 days of diagnosis, 3564 of 6945 (51.3%) underwent modified radical mastectomy without immediate reconstruction, and 4395 of 6945 (63.3%) appropriately received radiotherapy after mastectomy. Receipt of GCC was more likely among patients aged 60 to 69 years vs those aged 40 to 49 years (odds ratio [OR], 1.26; 95% CI, 1.03-1.54) and patients with nodal involvement vs those who did not have nodal involvement (eg, cN1: OR, 1.38; 95% CI, 1.15-1.67) (both *P* < .001) (Table 2). Receipt of GCC was least likely among patients with the hormone receptor–positive/*ERBB2*-positive biomarker subtype (vs hormone receptor–positive/*ERBB2*-negative: OR, 0.41; 95% CI, 0.33-0.51) and less likely among patients treated in 2014-2018 vs those treated in 2010-2013 (OR, 0.63; 95% CI, 0.55-0.72) (both *P* < .001).

**Table 2.** Multivariable Regression Analysis for Likelihood of Guideline-Concordant Care Receipt^a^

Variable	OR (95% CI)	*P* value
Age range, y		
40-49	1 [Reference]	<.001
50-59	1.06 (0.89-1.27)	
60-69	1.26 (1.03-1.54)	
≥70	0.75 (0.58-0.98)	
Clinical nodal category		
N0	1 [Reference]	<.001
N1	1.38 (1.15-1.67)	
N2	1.28 (1.02-1.61)	
N3	1.18 (0.93-1.50)	
Year of treatment		
2010-2013	1 [Reference]	<.001
2014-2018	0.63 (0.55-0.72)	
Tumor subtype		
Hormone receptor–positive/*ERBB2*-negative	1 [Reference]	<.001
Hormone receptor–negative/*ERBB2*-positive	1.01 (0.85-1.21)	
Hormone receptor–positive/*ERBB2*-positive	0.41 (0.33-0.51)	
Triple negative	0.95 (0.81-1.12)	

Abbreviation: OR odds ratio.

^a^ The model also included race and ethnicity, grade, insurance status, hospital location, and distance from hospital, but only variables significant on univariate and multivariable analysis are included in the table.

The likelihood of overall GCC receipt did not differ significantly by patient race and ethnicity, insurance status, location, or histologic characteristics, but there were disparities for modality-specific GCC. Asian (OR, 0.48; 95% CI, 0.27-0.84), Black (OR, 0.53; 95% CI, 0.41-0.68), and Hispanic (OR, 0.40; 95% CI, 0.29-0.55) patients with IBC were less likely to initiate NST within 60 days of diagnosis compared with White patients (*P* < .001) (eTable 1 in Supplement 1). Patients with hormone receptor–negative/*ERBB2*-positive (OR, 1.39; 95% CI, 1.06-1.84) and triple-negative (OR, 1.88; 95% CI, 1.45-2.45) subtypes vs hormone receptor–positive/*ERBB2*-negative subtypes and those with private insurance vs uninsured (OR, 1.87; 95% CI, 1.26-2.78) were more likely to initiate NST within 60 days of diagnosis (all *P* < .001).

Guideline-concordant surgery was performed in 51.3% (3564 of 6945) of the patients. A total of 79.4% (4332 of 5455) of the patients underwent axillary lymph node dissection, while 20.6% (1123 of 5455) of the patients did not undergo axillary lymph node dissection, instead undergoing sentinel lymph node biopsy or no axillary surgery. Patients aged 50 years or older were more likely to undergo guideline-concordant surgery than patients younger than 50 years (eg, age 60-69 vs 40-49 years: OR, 1.26; 95% CI, 1.03-1.54; *P* < .001). Likewise, patients with a higher clinical nodal burden (cN1-3 vs cN0) were more likely to undergo axillary lymph node dissection (eg, cN1 vs cN0: OR, 1.38; 95% CI, 1.15-1.67; *P* = .006) (eTable 2 in Supplement 1). Patients in urban compared with metropolitan locations were more likely to be treated with guideline-concordant surgery (OR, 1.29; 95% CI, 1.09-1.52; *P* = .01). In addition, patients treated in a more contemporary cohort (2014-2018) were less likely to undergo guideline-concordant surgery vs patients treated earlier (2010-2013) (OR, 0.82; 95% CI, 0.73-0.93; *P* < .001). Of note, 35.6% (1966 of 5530) of patients undergoing mastectomy underwent immediate breast reconstruction despite it not being recommended for patients with IBC. Radiotherapy was omitted or administered before surgery in 36.7% (2550 of 6945) of the patients. Black race, age 70 years or older, treatment in a more contemporary setting (2014-2018), and triple-negative subtypes were all associated with a lower likelihood of receiving radiotherapy in the proper sequence (eTable 3 in Supplement 1). Patients with private/managed care vs uninsured patients (OR, 2.12; 95% CI, 1.57-2.87; *P* < .001), pathologic complete response vs no pathologic complete response (OR, 2.00; 95% CI, 1.61-2.48; *P* < .001), and higher clinical nodal category (cN1-3) vs cN0 (OR, 1.33; 95% CI, 1.06-1.67), cN2 (OR, 1.31; 95% CI, 1.06-1.64), and cN3 (OR, 1.33; 95% CI, 1.06-1.67) (*P* = .006) were more likely to receive radiotherapy in the proper sequence.

### Overall Survival

**Figure 1.**
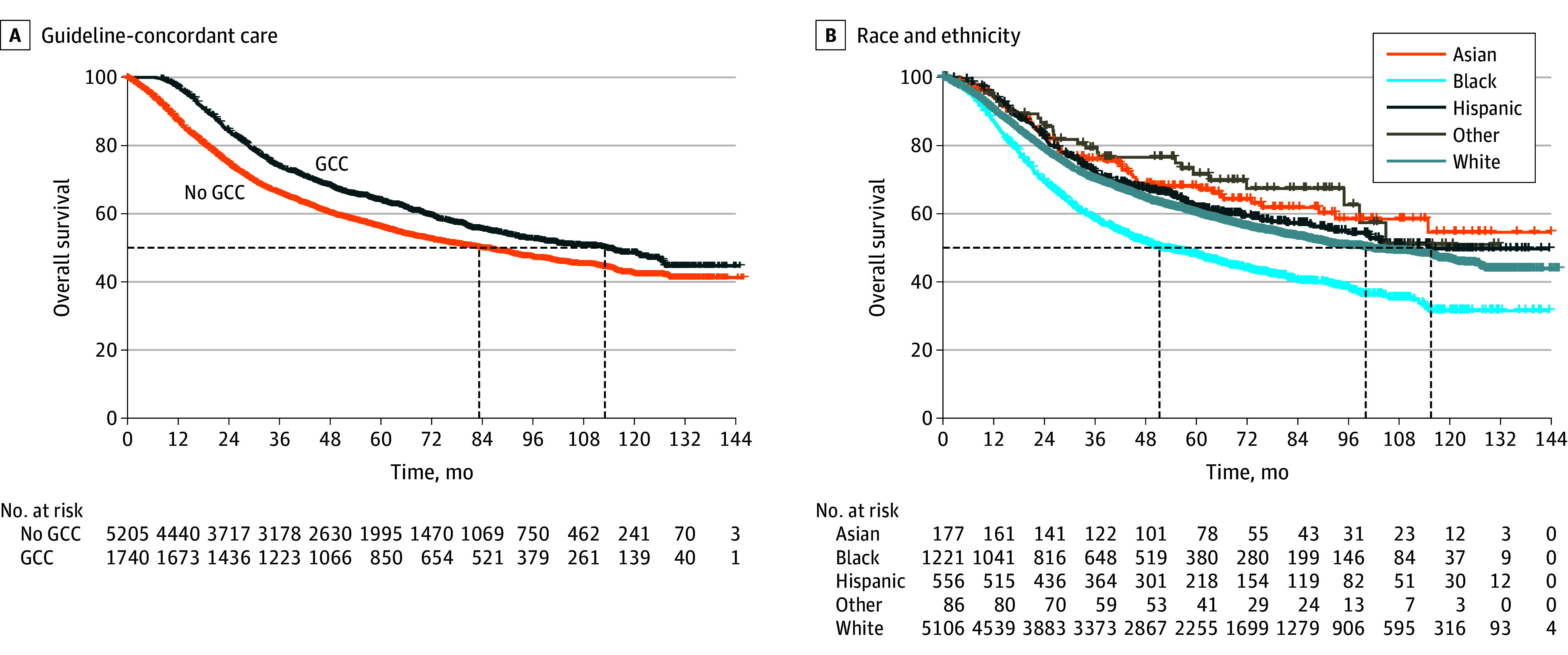
Overall Survival by Receipt of Guideline-Concordant Care (GCC) and Race and Ethnicity Receipt of GCC (A) and race and ethnicity (B), using Kaplan-Meier analysis, among women with nonmetastatic inflammatory breast cancer; National Cancer Database, 2010-2018. The dashed lines indicate visual emphasis for the curves.

**Figure 2.**
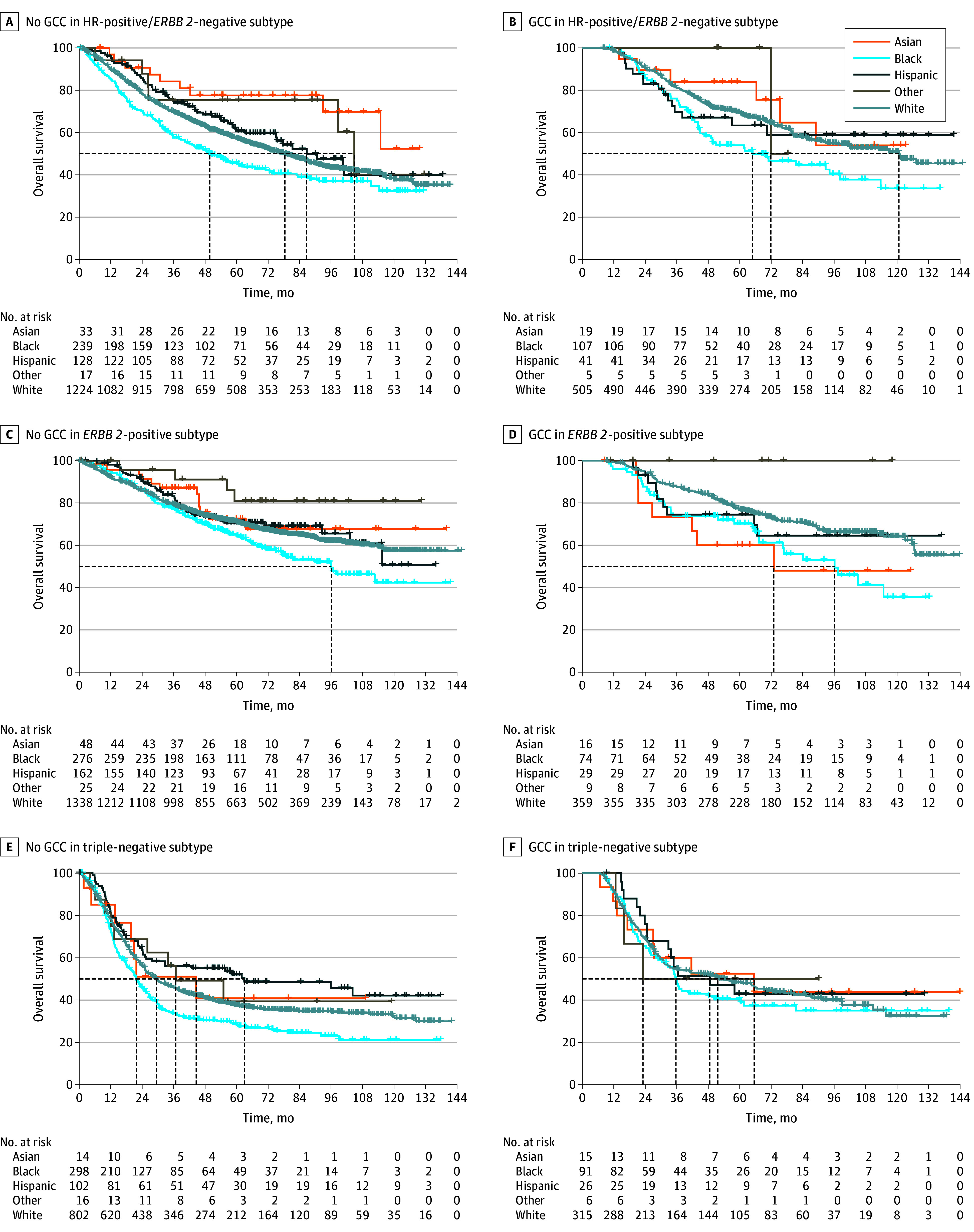
Overall Survival by Race, Tumor Subtype, and Guideline-Concordant Care (GCC) Receipt Receipt of GCC in hormone receptor (HR)–negative/*ERBB2*-negative subtypes not receiving GCC (A), HR–positive/*ERBB2*-negative subtypes receiving GCC (B), *ERBB2*-positive subtypes not receiving GCC (C), *ERBB2*-positive subtypes receiving GCC (D), triple-negative subtypes not receiving GCC (E), and triple-negative subtypes receiving GCC (F), using Kaplan-Meier analysis, among women with nonmetastatic inflammatory breast cancer; National Cancer Database, 2010-2018. The dashed lines indicate visual emphasis for the curves.

Overall and among all subtypes, patients receiving GCC had improved OS compared with patients who did not receive GCC, with an unadjusted 5-year OS rate of 63.9% (95% CI, 61.6%-66.3%) in patients with vs 55.9% (95% CI, 54.6%-57.4%) of those without GCC (Figure 1A). Unadjusted OS at 5 and 10 years was lowest among Black patients compared with Asian and Pacific Islander, Hispanic, and White patients (Figure 1B), and this trend was observed across all subtypes (eFigure 2 in Supplement 1). The 5-year OS rate for Black patients was 47.9% compared with 60.0% among White patients. Among patients with triple-negative breast cancer who did not receive GCC, Black patients had significantly lower survival compared with Asian and White patients, with Black patients having a 5-year survival rate of 28.8% compared with a 5-year survival rate of 37.4% among White patients. However, among patients with triple-negative breast cancer who received GCC, survival outcomes did not differ significantly by race and ethnicity (Figure 2). Survival remained lowest for Black patients with hormone receptor–positive/*ERBB2*-negative or hormone receptor–negative/*ERBB2*-positive cancer and was not affected by GCC receipt.

On multivariable analysis, GCC receipt was associated with improved adjusted OS (hazard ratio [HR], 0.75; 95% CI, 0.68-0.84; *P* < .001) (Table 3). Black compared with White race (HR, 1.41; 95% CI, 1.26-1.58), triple-negative subtype vs HR-positive/*ERBB2*-negative (HR, 1.61; 95% CI, 1.44-1.80), clinical nodal involvement vs cN0 (cN2: HR, 1.21; 95% CI, 1.04-1.40, and cN3: HR, 1.41; 95% CI, 1.21-1.64), and age 70 years or older (HR, 2.04; 95% CI, 1.71-2.44) were independently associated with lower adjusted OS (all *P* < .001). Private/managed care vs being uninsured (HR, 0.63; 95% CI, 0.51-0.77) or having other government insurance (HR, 0.57; 95% CI, 0.36-0.91) (both *P* < .001) and being treated at an academic research program vs a community cancer program (HR, 1.21; 95% CI, 1.02-1.43; *P* = .03) were associated with better survival. An interaction term between race and ethnicity and GCC was assessed but was not found to be significant.

**Table 3.** Multivariable Cox Proportional Hazards Model for Overall Survival, Women With Nonmetastatic Inflammatory Breast Cancer, National Cancer Database, 2010-2018^a^

Variable	Hazard ratio (95% CI)	*P* value
GCC	0.75 (0.68-0.84)	<.001
Subtype		
Hormone receptor–positive/*ERBB2*-negative	1 [Reference]	<.001
Hormone receptor–negative/*ERBB2*-positive	0.69 (0.60-0.79)	
Hormone receptor–positive/*ERBB2*-positive	0.65 (0.57-0.75)	
Triple negative	1.61 (1.44-1.80)	
Age, y		
40-49	1 [Reference]	<.001
50-59	1.13 (1.0-1.29)	
60-69	1.21 (1.04-1.40)	
≥70	2.04 (1.71-2.44)	
Race		
Asian or Pacific Islander	0.73 (0.51-1.05)	<.001
Black	1.41 (1.26-1.58)	
Hispanic	0.85 (0.70-1.02)	
White	1 [Reference]	
Other^b^	0.73 (0.43-1.25)	
Grade		
I	1 [Reference]	<.001
2	1.33 (0.98-1.80)	
3	1.53 (1.13-2.08)	
Clinical nodal category		
N0	1 [Reference]	<.001
N1	0.94 (0.83-1.07)	
N2	1.21 (1.04-1.40)	
N3	1.41 (1.21-1.64)	
Insurance		
Uninsured	1 [Reference]	<.001
Medicaid	0.86 (0.69-1.07)	
Medicare	0.81 (0.65-1.02)	
Other government	0.57 (0.36-0.91)	
Private/managed care	0.63 (0.51-0.77)	
Facility		
Academic research program	1 [Reference]	.03
Community cancer program	1.21 (1.02-1.43)	
Comprehensive community cancer program	1.15 (1.04-1.28)	
Integrated network cancer program	1.11 (0.97-1.27)	

Abbreviation: GCC, guideline-concordant care.

^a^ The model also included year of treatment, hospital location, and distance from hospital, but only variables significant on univariate and multivariable analysis are included in the table.

^b^ Other includes Aleutian or Eskimo, American Indian, Chamorran, Fiji, Guamanian, Hawaiian, Melanesian, Micronesian, New Guinean, Polynesian, Samoan, Tahitian, and Tongan.

## Discussion

Guideline-concordant care for IBC is uncommon, with only 25.1% of patients receiving GCC in this study cohort. Furthermore, the use of GCC decreased over time, with 45% of patients receiving GCC between 2010 and 2012 compared with only 22% of patients between 2016 and 2018. Despite this trend, GCC has been associated with improved OS in numerous studies.^6,19,20,21,22^ While most patients (91%) received NST within 60 days of diagnosis, only 51% of patients underwent modified radical mastectomy without reconstruction and only 63% received postmastectomy radiotherapy in the correct sequence, suggesting that as systemic therapy improves there is a trend toward locoregional therapy deescalation, despite insufficient evidence to support doing so for this aggressive subtype of breast cancer.

Advances in systemic therapy have improved survival outcomes for patients with both non-IBC and IBC.^23,24^ However, survival for IBC remains significantly lower than for non-IBC.^25^ Response to NST varies widely by subtype and poor response to NST portends a worse prognosis.^26,27,28^ In a study examining all patients with nonmetastatic IBC treated from 2010 to 2015 in the National Cancer Database, 5-year OS rates ranged from 44.0% in patients with estrogen receptor (ER)-negative/*ERBB2*-negative to 64.9% in ER-positive/*ERBB2*-negative and 74.0% in *ERBB2*-positive IBC.^29^ Similarly, pathologic complete response rates for patients with IBC vary by tumor subtype, and rates of pathologic complete response are significantly lower for patients with IBC compared with non-IBC.^21,30^ For patients with *ERBB2*-positive breast cancer, rates of pathologic complete response range from 15% to 49%.^31^ In our study, GCC receipt was lowest among patients with hormone receptor–positive/*ERBB2*-positive, likely due to high rates of pathologic complete response among patients with *ERBB2*-positive compared with those with hormone receptor–positive*/ERBB2*-negative IBC and the opportunity for adjuvant treatment with endocrine therapy. Improvements in survival and pathologic complete response have likely resulted in decreased receipt of guideline-concordant surgery and radiotherapy.

Delays to initiation of NST of more than 61 days have been associated with an increased risk of death from breast cancer.^32^ Among patients receiving NST in our study, 9% experienced a delay to initiation of NST of more than 60 days. On multivariable analysis, Asian (OR, 0.48; 95% CI, 0.27-0.84), Black (OR, 0.53; 95% CI, 0.41-0.68), and Hispanic (OR, 0.40; 95% CI, 0.29-0.55) patients were less likely than White patients to receive NST within 60 days of diagnosis. Timely initiation of chemotherapy, particularly among patients with the triple-negative subtype, could play a role in survival disparities by race and ethnicity. Black patients experienced the lowest OS in our study, but racial and ethnic disparities in OS were not observed among patients with triple-negative IBC receiving GCC, suggesting that timely, routinized receipt of NST could potentially reduce racial and ethnic disparities in survival outcomes. While survival outcomes for Black women improved among all tumor subtypes when GCC was given, racial and ethnic disparities in survival outcomes persisted among patients with hormone receptor–positive/*ERBB2*-negative and hormone receptor–positive/*ERBB2*-positive subtypes. Systemic racism and other barriers to care are likely at play and will need to be the targets of intervention as Black women with IBC have been reported to have comparable levels of treatment adherence as White women.^33^ As triple-negative IBC outcomes improve with increased use of pembrolizumab, we must guard against the introduction of racial and ethnic disparity where there previously appeared to be none.

Use of immediate breast reconstruction has been increasing.^10,11,12,13^ Hoffman and colleagues^11^ found increasing rates of immediate breast reconstruction from 6.3% in 2004 to 10.1% in 2016, without prospective evidence supporting its oncologic safety. In our study, 35.6% (1966 of 5530) of patients undergoing mastectomy underwent immediate breast reconstruction, despite the potential for adjuvant treatment delays and no evidence of oncologic safety for immediate breast reconstruction after IBC diagnosis.^34,35,36^

Axillary lymph node dissection is the standard of care for patients with IBC, as the rate of nodal positivity at presentation has been shown to be as high as 92%. Fayanju et al^37^ reported that, even among patients with cN0 cancer, as many as 50% were node-positive at the time of surgery after NST. A few small studies have examined the feasibility and accuracy of sentinel lymph node biopsy^38,39,40^ after NST administration and documented unacceptably high false-negative rates. Despite a lack of oncologic safety data for sentinel lymph node biopsy in this population, the use of sentinel lymph node biopsy for patients with IBC is increasing.^41^ Twenty-one percent of the women in this study did not undergo axillary lymph node dissection. Patients with a higher clinical nodal burden (cN1-2 vs cN0) were more likely to undergo axillary lymph node dissection (*P* = .006).

In a retrospective review of a prospectively maintained single-institution database, 453 patients with nonmetastatic IBC were examined to evaluate the frequency and factors associated with pathologic node-negativity (ypN0).^42^ Overall, 34% (156 of 453) were ypN0 after NST; ycN0 status and hormone receptor–negative/*ERBB2*-positive subtype were associated with nodal pathologic complete response (ypN0). In a study of 3471 women identified from the National Cancer Database with nonmetastatic IBC, the extent of axillary surgery (>10 lymph nodes removed) was not associated with improved adjusted OS for patients with cN0 nodal category, again despite lymph node involvement in half of the patients at surgery.^37^ These data suggest that there may be a group of patients with IBC in whom sentinel lymph node biopsy may be safe, and there are currently 2 open protocols^43,44^ examining the feasibility and accuracy of sentinel lymph node biopsy in patients with IBC. However, as we await data from these trials, axillary lymph node dissection remains the standard of care for patients with IBC.

Radiotherapy has been associated with a survival benefit among patients with IBC.^45,46^ However, a significant proportion of patients (37%) did not receive PMRT in our study. On multivariable analysis, age 70 years or older, Black race, receipt of treatment in a later time point (2014-2018), and triple-negative subtype were all associated with a lower likelihood of receiving guideline-concordant PMRT. Patients with private insurance/managed care and higher clinical nodal status (cN1-3 vs cN0) were more likely to receive radiotherapy in the proper sequence. Our findings are similar to those of another study that examined 8273 women from 1998 to 2011 identified in the National Cancer Database.^47^ Overall, 30.3% of the patients did not receive PMRT. They also found that patients with private insurance and higher clinical nodal category were more likely to receive PMRT. These findings suggest that clinicians are deescalating the use of PMRT for patients with more limited nodal disease at presentation and that the type of insurance coverage may present a barrier to receipt of PMRT.

Increasing age and triple-negative tumor subtype were associated with lower OS. Age older than 70 years was also associated with decreasing GCC receipt, a trend that has been reported in other studies.^48,49^ Drapalik and colleagues^48^ examined GCC receipt by age and found that patients older than 65 years were significantly more likely to have NST omitted as well as modified radical mastectomy without immediate reconstruction and PMRT compared with younger patients. However, GCC receipt was associated with improved OS among patients older than 65 years. Similarly, Adesoye et al^49^ examined 523 patients with IBC treated at a single institution by a multidisciplinary team from 2010 to 2019. They found no significant differences in treatment patterns by age or clinical outcomes when comparing patients aged 40 years or younger with those aged 65 years or older. This suggests that age alone may not predispose patients to worse outcomes and that shared decision-making and multidisciplinary treatment may improve adherence and overall outcomes.

### Limitations

This study has limitations. While the National Cancer Database is one of the largest cancer registries in the world, there is some missingness among the data as patients do not always complete their care within a single institution, which could lead to underrepresentation of some groups. Furthermore, there is no central review of clinical diagnoses, and IBC can be misclassified as other forms of locally advanced breast cancer. There is also no breast cancer–specific survival outcome available within the dataset, but given the aggressive nature of IBC, OS was the primary outcome of our analysis and a reasonable surrogate. In addition, data regarding progression while receiving therapy are not available in the National Cancer Database. Patients who did not complete systemic therapy or radiotherapy were excluded from this study, as this may have been due to disease progression, representing an additional limitation of our study. Despite these limitations, to our knowledge, this study is one of the largest to date that examines trends and disparities in the delivery of GCC for IBC and its association with survival. Future work to understand the barriers and facilitators of GCC is needed to determine the next steps to improving disparities in care for patients with IBC.

## Conclusions

In this cohort study of women with nonmetastatic IBC, GCC was associated with greater OS. However, most patients with IBC do not appear to receive GCC and the rates of GCC have decreased in more recent years. While OS for IBC was lowest among Black women, there was no racial and ethnic disparity among GCC recipients with triple-negative disease. These findings suggest that timely GCC receipt can improve OS among patients with IBC and may help to mitigate racial and ethnic disparities in OS among patients with IBC.

## Notes

Supplement 1. **eFigure 1.** Decreasing Frequency of Guideline-Concordant Care (GCC) Over Time Among Women With Non-Metastatic Inflammatory Breast Cancer, National Cancer Database, 2010-2018 **eFigure 2.** Overall Survival by Race Among Women With Non-Metastatic Inflammatory Breast Cancer Using Kaplan-Meier Method, National Cancer Database, 2010-2018 **eTable 1.** Multivariable Regression Analysis for Likelihood of Neoadjuvant Systemic Therapy Within 60 days of Diagnosis, Women With Non-Metastatic Inflammatory Breast Cancer, National Cancer Database, 2010-2018 **eTable 2.** Multivariable Regression Analysis for Likelihood of Guideline-Concordant Surgery, Women With Non-Metastatic Inflammatory Breast Cancer, National Cancer Database, 2010-2018 **eTable 3.** Multivariable Regression Analysis for Likelihood of Guideline-Concordant Postmastectomy Radiation, Women With Non-Metastatic Inflammatory Breast Cancer, National Cancer Database, 2010-2018

Supplement 2.
Data Sharing Statement
