# Supplementary material for: Trends in Guideline-Concordant Care for Inflammatory Breast Cancer
Source: JAMA Netw Open. 2025 Feb 26;8(2):e2454506. doi: 10.1001/jamanetworkopen.2024.54506 (PMC11866030; doi:10.1001/jamanetworkopen.2024.54506)
Supplement: Supplement 1. — eFigure 1. Decreasing Frequency of Guideline-Concordant Care (GCC) Over Time Among Women With Non-Metastatic Inflammatory Breast Cancer, National Cancer Database, 2010-2018 eFigure 2. Overall Survival by Race Among Women With Non-Metastatic Inflammatory Breast Cancer Using Kaplan-Meier Method, National Cancer Database, 2010-2018 eTable 1. Multivariable Regression Analysis for Likelihood of Neoadjuvant Systemic Therapy Within 60 days of Diagnosis, Women With Non-Metastatic Inflammatory Breast Cancer, National Cancer Database, 2010-2018 eTable 2. Multivariable Regression Analysis for Likelihood of Guideline-Concordant Surgery, Women With Non-Metastatic Inflammatory Breast Cancer, National Cancer Database, 2010-2018 eTable 3. Multivariable Regression Analysis for Likelihood of Guideline-Concordant Postmastectomy Radiation, Women With Non-Metastatic Inflammatory Breast Cancer, National Cancer Database, 2010-2018 [file jamanetwopen-e2454506-s001.pdf]

Supplementary Online Content

Tadros A, Diskin B, Sevilimedu V, et al. Trends in guideline-concordant care for inflammatory breast cancer. *JAMA Netw Open*. 2025;8(2):e2454506. doi:10.1001/jamanetworkopen.2024.54506

eFigure 1. Decreasing Frequency of Guideline-Concordant Care (GCC) Over Time Among Women With Non-Metastatic Inflammatory Breast Cancer, National Cancer Database, 2010-2018

eFigure 2. Overall Survival by Race Among Women With Non-Metastatic Inflammatory Breast Cancer Using Kaplan Meier Method, National Cancer Database, 2010-2018

eTable 1. Multivariable Regression Analysis for Likelihood of Neoadjuvant Systemic Therapy Within 60 days of Diagnosis, Women With Non-Metastatic Inflammatory Breast Cancer, National Cancer Database, 2010-2018

eTable 2. Multivariable Regression Analysis for Likelihood of Guideline-Concordant Surgery, Women With Non-Metastatic Inflammatory Breast Cancer, National Cancer Database, 2010-2018

eTable 3. Multivariable Regression Analysis for Likelihood of Guideline-Concordant Postmastectomy Radiation, Women With Non-Metastatic Inflammatory Breast Cancer, National Cancer Database, 2010-2018

This supplementary material has been provided by the authors to give readers additional information about their work.

© 2025 Tadros A et al. *JAMA Network Open.*

eFigure 1. Decreasing Frequency of Guideline-Concordant Care (GCC) over Time among Women with Non-Metastatic Inflammatory Breast Cancer, National Cancer Database, 2010-2018

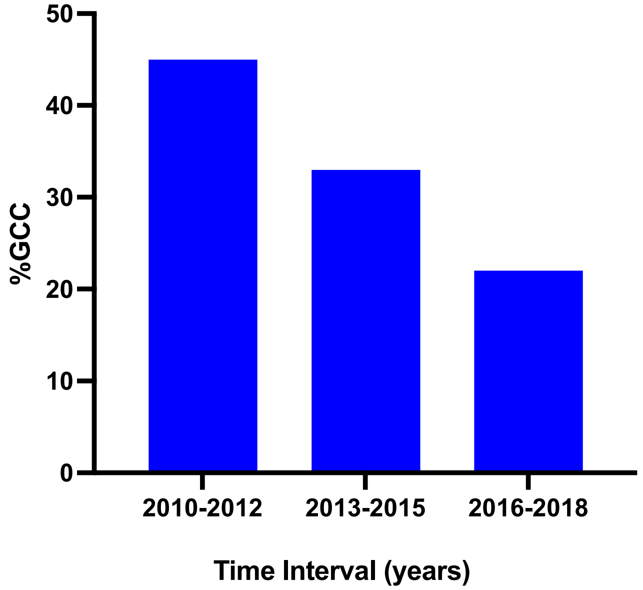

© 2025 Tadros A et al. *JAMA Network Open.*

eFigure 2. Overall Survival by Race among Women with Non-Metastatic Inflammatory Breast Cancer using Kaplan Meier method, National Cancer Database, 2010-2018 (a) HR+ HER2- subtype; (b) HER2+ subtype (c) Triple-Negative subtype

(a)

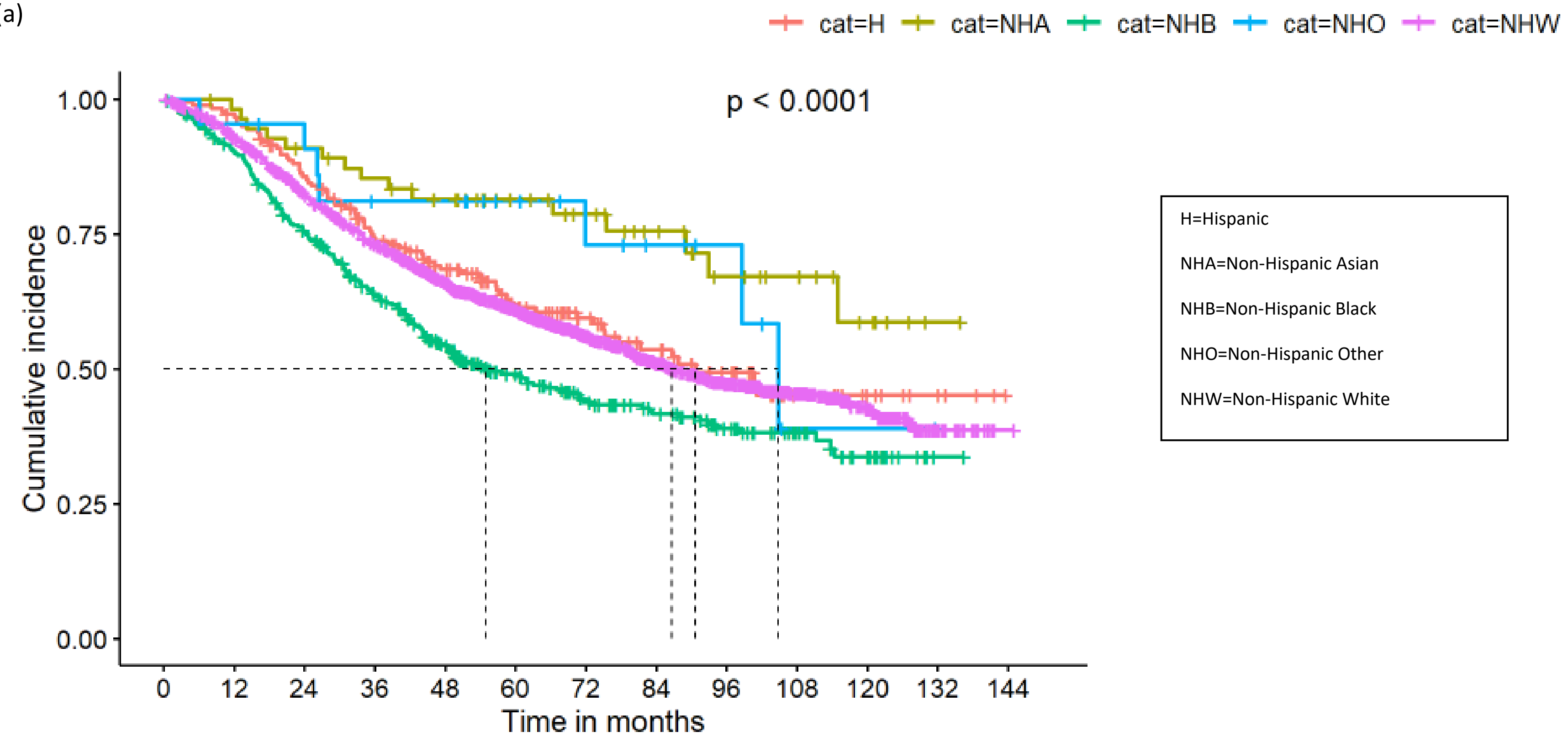

H=Hispanic

NHA=Non-Hispanic Asian

NHB=Non-Hispanic Black

NHO=Non-Hispanic Other

NHW=Non-Hispanic White

© 2025 Tadros A et al. *JAMA Network Open.*

(b)

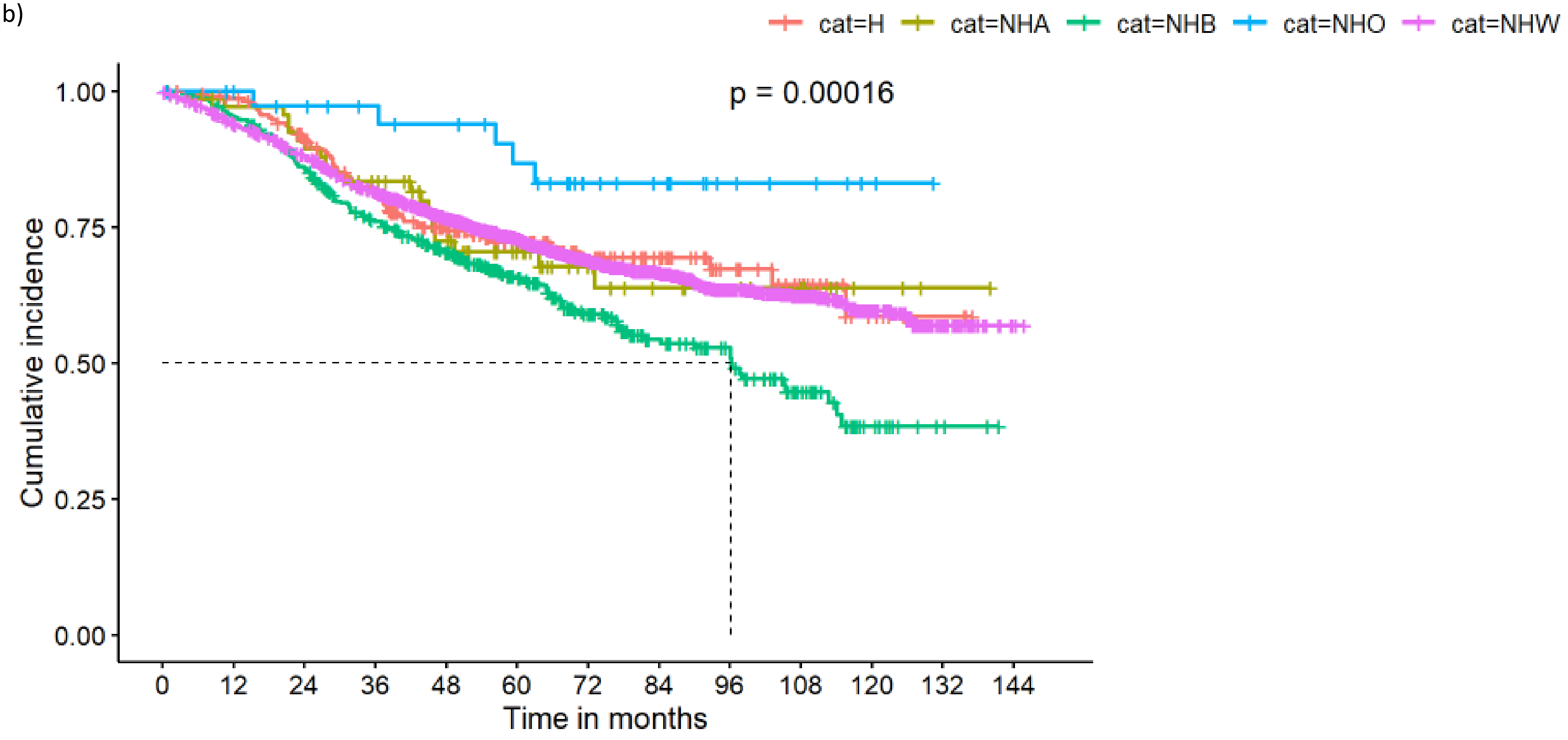

© 2025 Tadros A et al. *JAMA Network Open.*

(c)

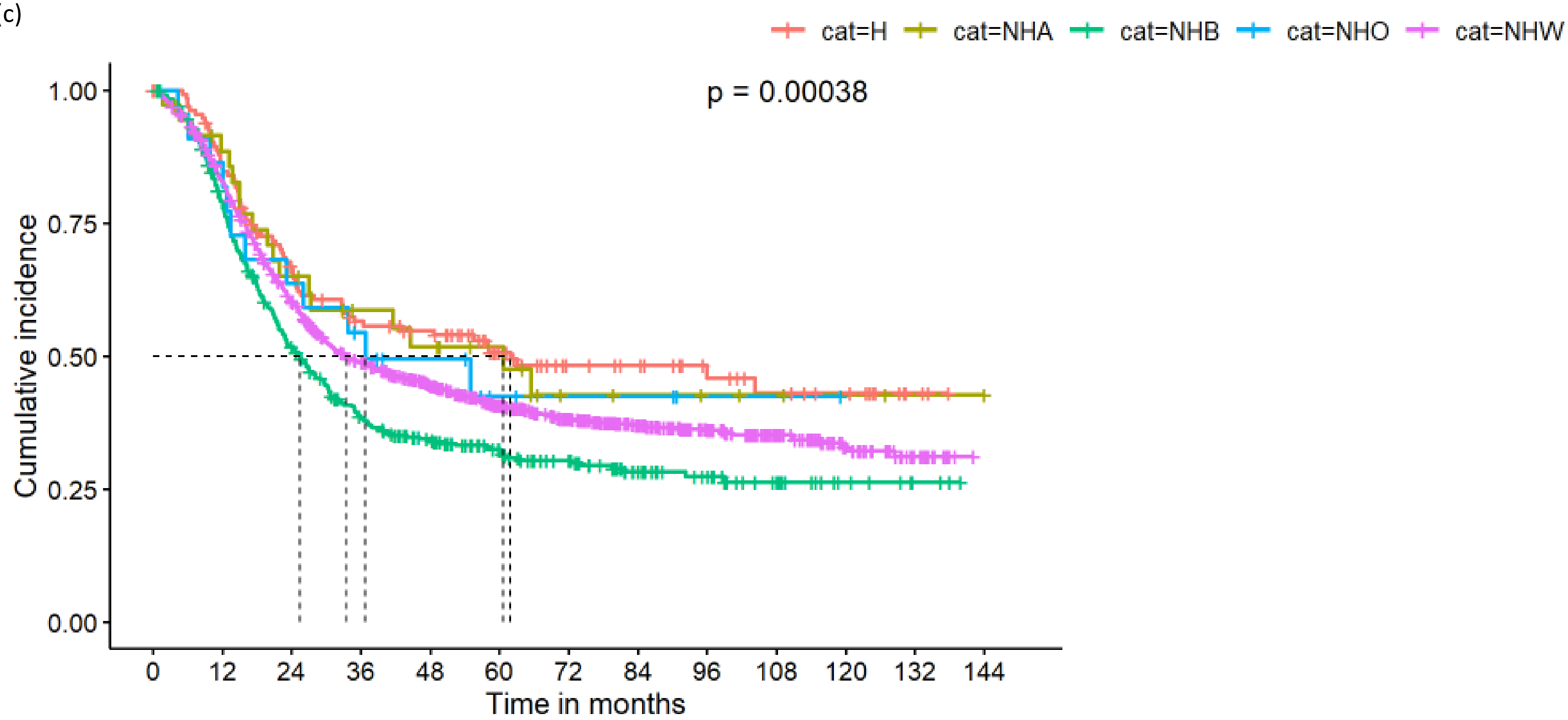

© 2025 Tadros A et al. *JAMA Network Open.*

eTable1. Multivariable regression analysis for Likelihood of Neoadjuvant Systemic Therapy within 60 days of Diagnosis, Women with Non-Metastatic Inflammatory Breast Cancer, National Cancer Database, 2010-2018

	OR 95% CI	p-value
**Race/Ethnicity**		<0.001
White	ref	
Asian/Pacific Islander	0.48 (0.27, 0.84)	
Black	0.53 (0.41, 0.68)	
Hispanic	0.4 (0.29, 0.55)	
Other	0.49 (0.23, 1.04)	
**Insurance**		<0.001
Uninsured	ref	
Medicaid	1.17 (0.77, 1.78)	
Medicare	1.41 (0.94, 2.12)	
Other government	0.9 (0.43, 1.89)	
Private/Managed Care	1.93 (1.30, 2.85	
**Tumor Subtype**		<0.001
HR+HER2-	ref	
HR-HER2+	1.39 (1.06, 1.84)	
HR+HER2+	1.16 (0.88, 1.52)	
TN	1.88 (1.45, 2.45)	

© 2025 Tadros A et al. *JAMA Network Open.*

eTable2. Multivariable regression analysis for Likelihood of Guideline-Concordant Surgery, Women with Non-Metastatic Inflammatory Breast Cancer, National Cancer Database, 2010-2018

	OR 95% CI	p-value
**Age**		<0.001
40-49	ref	
50-59	1.29 (1.10, 1.50)	
60-69	1.43 (1.19, 1.71)	
≥70	1.54 (1.20, 1.97)	
**Clinical Nodal Stage**		<0.001
N0	ref	
N1	1.39 (1.18, 1.64)	
N2	1.48 (1.20, 1.83)	
N3	1.31 (1.06, 1.63)	
**Year of Treatment**		<0.001
2010-2013	ref	
2014-2018	0.82 (0.73, 0.93)	
**Location**		0.012
Metro	ref	
Rural	1.15 (0.77, 1.73)	
Urban	1.29 (1.09, 1.52)	

© 2025 Tadros A et al. *JAMA Network Open.*

eTable3. Multivariable regression analysis for Likelihood of Guideline-Concordant Postmasectomy Radiation, Women with Non-Metastatic Inflammatory Breast Cancer, National Cancer Database, 2010-2018

	OR 95% CI	p-value
**Age**		<0.001
40-49	ref	
50-59	0.92 (0.77, 1.10)	
60-69	0.94 (0.76, 1.15)	
≥70	0.40 (0.31, 0.52)	
**Race/Ethnicity**		<0.001
White	ref	
Asian/Pacific Islander	1.07 (0.84, 1.41)	
Black	0.73 (0.62, 0.87)	
Hispanic	1.09 (0.84, 1.41)	
Other	0.65 (0.35, 1.20)	
**Clinical Nodal Stage**		<0.001
N0	ref	
N1	1.36 (1.14, 1.62)	
N2	1.31 (1.06, 1.64)	
N3	1.33 (1.06, 1.67)	
**Year of Treatment**		<0.001
2010-2013	ref	
2014-2018	0.85 (1.06, 1.67)	
**Insurance**		<0.001
Uninsured	ref	
Medicaid	1.25 (0.90, 1.74)	
Medicare	1.39 (0.99, 1.94)	
Other government	1.63 (0.88, 3.02)	
Private/Managed Care	2.12 (1.57, 2.87)	
**Tumor Subtype**		<0.001
HR+HER2-	ref	
HR-HER2+	0.97 (0.81, 1.17)	
HR+HER2+	1.10 (0.91, 1.33)	
TN	0.80 (0.67, 0.94)	

© 2025 Tadros A et al. *JAMA Network Open.*
