# Supplementary material for: Trends in Guideline-Concordant Care for Inflammatory Breast Cancer
Source: JAMA Netw Open. 2025 Feb 26;8(2):e2454506. doi: 10.1001/jamanetworkopen.2024.54506 (PMC11866030; doi:10.1001/jamanetworkopen.2024.54506)
Supplement: Supplement 2. — Data Sharing Statement [file jamanetwopen-e2454506-s002.pdf]

Data Sharing Statement

Tadros. Trends in Guideline-Concordant Care for Inflammatory Breast Cancer. *JAMA Netw Open*. Published February 11, 2025. doi:10.1001/jamanetworkopen.2024.54506

Data
Data available: Yes
Data types: Other (please specify)
Additional Information: Data used for these analyses are publicly available via the American College of Surgeons (ACS) upon request to the ACS for access to the National Cancer Database (NCDB).
How to access data: https://www.facs.org/media/xtvknrsu/2020-puf-instructions-to-potential-applicants.pdf
When available: With publication

Supporting Documents
Document types: None

Additional Information
Who can access the data: Anyone from a Commission on Cancer (CoC)-accredited program
Types of analyses: Code can be provided on request to the statistician.
Mechanisms of data availability: Per the ACS
